# Local field potentials reflect multiple spatial scales in V4

**DOI:** 10.3389/fncom.2013.00021

**Published:** 2013-03-26

**Authors:** Patrick J. Mineault, Theodoros P. Zanos, Christopher C. Pack

**Affiliations:** Department of Neurology and Neurosurgery, Montreal Neurological Institute, McGill UniversityMontreal, QC, Canada

**Keywords:** receptive field, multiunit activity, local field potentials, V4, visual cortex

## Abstract

Local field potentials (LFP) reflect the properties of neuronal circuits or columns recorded in a volume around a microelectrode (Buzsáki et al., 2012). The extent of this integration volume has been a subject of some debate, with estimates ranging from a few hundred microns (Katzner et al., 2009; Xing et al., 2009) to several millimeters (Kreiman et al., 2006). We estimated receptive fields (RFs) of multi-unit activity (MUA) and LFPs at an intermediate level of visual processing, in area V4 of two macaques. The spatial structure of LFP receptive fields varied greatly as a function of time lag following stimulus onset, with the retinotopy of LFPs matching that of MUAs at a restricted set of time lags. A model-based analysis of the LFPs allowed us to recover two distinct stimulus-triggered components: an MUA-like retinotopic component that originated in a small volume around the microelectrodes (~350 μm), and a second component that was shared across the entire V4 region; this second component had tuning properties unrelated to those of the MUAs. Our results suggest that the LFP reflects neural activity across multiple spatial scales, which both complicates its interpretation and offers new opportunities for investigating the large-scale structure of network processing.

## Introduction

Local field potentials (LFP) are low-frequency oscillations in the extracellular electric potential detectable through microelectrode recordings. Although they reflect a variety of electrical phenomena, the total synaptic current in a volume around the microelectrode is the major contributor to LFPs (Buzsáki et al., 2012). They thus offer a complementary signal to spikes, reflecting subthreshold network activity at larger spatial and longer temporal scales than are accessible through single unit recording.

The nature of the relationship between spikes and LFPs is a controversial issue that has attracted much interest (Bauer et al., 1995; Henrie and Shapley, 2005; Mukamel et al., 2005; Kreiman et al., 2006; Liu and Newsome, 2006; Nir et al., 2007; Belitski et al., 2008; Gieselmann and Thiele, 2008; Rasch et al., 2008; Katzner et al., 2009; Khawaja et al., 2009; Xing et al., 2009; Eggermont et al., 2011; Hwang and Andersen, 2011; Jia et al., 2011; Liebe et al., 2011; Lindén et al., 2011; Tsui and Pack, 2011; Zanos et al., 2011b; Lashgari et al., 2012). In a typical experimental scenario, a well-understood property of multi-unit activity (MUA) is compared and contrasted with that of the LFP. For instance, it has been established, by comparing the orientation tuning of V1 MUA and LFP activity, that the LFP activity in V1 could result from the spiking activity in a small volume around the electrode, on the order of 250 μm (Katzner et al., 2009). Xing et al. (2009) came to a similar estimate by comparing the size of MUA and LFP receptive fields (RFs) in V1. On the other hand, Kreiman et al. (2006) found that selectivity of LFPs for objects is best explained by hypothesizing an integration radius of a few millimeters.

The difference in the estimated integration radii across experiments may reflect the selection of different components of the LFP for analysis. For instance, the power in the high-frequency gamma band tends to be correlated with spiking activity (Ray and Maunsell, 2011) while the amplitude of the signal at lower frequencies has distinct tuning (Belitski et al., 2008); distinct components may have distinct integration properties (Berens et al., 2008). In addition, the physical size of the area under study, the regularity of its organization, and the correlation structure of its input (Lindén et al., 2011) can influence the properties of the LFP, and this may explain some of the discrepancies in the literature.

Many studies of the LFP have focused on V1 in particular, which is an unusual cortical area in that it is quite large, and it is known to have extremely precise columnar organization based on orientation selectivity (Ohki et al., 2005, 2006) and retinal position (Hubel and Wiesel, 1977; Blasdel and Fitzpatrick, 1984). Thus, LFPs in V1 may be unrepresentative of visual or sensory cortex as a whole. As a step toward understanding the LFP in cortex at large, we have recorded LFPs and MUAs in cortical area V4, a region that occupies an intermediate position in the visual hierarchy.

V4 recordings were carried out in two macaque monkeys, both of whom were implanted chronically with 96-electrode Utah arrays, which allowed us to relate the properties of the receptive fields to their physical location on the cortical surface. Using a sparse-noise stimulation procedure, we found that LFPs in V4 exhibit well-defined receptive fields whose positions change smoothly as a function of position on the cortical surface. However, a detailed analysis of the temporal properties of these signals revealed striking changes in RF position and size as a function of time following stimulus onset, such that the retinotopy of MUAs matched that of LFPs only at a restricted set of time lags.

These results could be explained by a model in which the LFP reflects multiple sources of input: a local, retinotopic input and a distant, shared input that had a similar effect across all of V4. By fitting such a model to our data, we found that the local input exhibited consistent retinotopy that approximated that of the simultaneously recorded MUAs. The shared input arrived at latencies that differed from those of the retinotopic input and that differed substantially between the two animals. These results suggest that the LFP reflects neural activity across multiple spatial scales, which both complicates its interpretation and offers new opportunities for investigating the large-scale structure of network processing.

## Results

### Preliminary analysis

**Figure 1:**
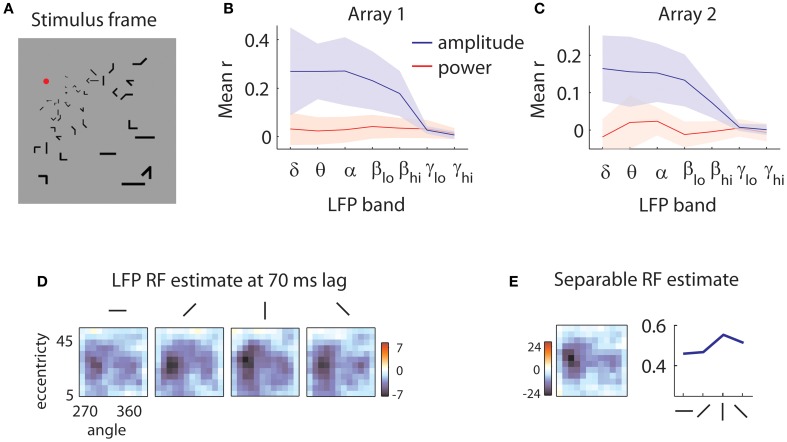
**Sample stimulus and receptive field. (A)** Sample stimulus frame. The stimulus was composed of sparse bars arranged on a log-polar grid to account for the magnification of the visual field with eccentricity. **(B)** Quality of reverse correlation fits to the amplitude of the LFPs (blue lines) and power (red lines) in different frequency bands for the first array. Delta: 0.5–4 Hz, theta: 4–8 Hz, alpha: 8–12 Hz, low beta: 12–20 Hz, high beta: 20–30 Hz, low gamma: 30–50 Hz, high gamma: 50–80 Hz. Quality of fit was evaluated by the correlation (r) of the predicted and measured responses in a validation dataset. Shaded error bars represent ±1 s.d. Power is not modulated by the stimulus; rather, the stimulus influences the low-frequency components of the amplitude of the LFPs. **(C)** Same as in **(B)**, for the second array. **(D)** Sample LFP RF estimate. The RF of the LFP is measured at a 70 ms time lag relative to stimulus onset. Each square represents the spatial RF for a given orientation. The shape of the spatial RF varies little with orientation. The color bar indicates peak *z*-values estimated through bootstrapping. Here, as in all subsequent RF illustrations, a Gaussian smoothing kernel with σ = 0.7 is applied. **(E)** Separable RF estimate. The RF shown in **(D)** is approximated as separable in space (left) and orientation (right). Little information is lost in the process, and *z*-values for the spatial envelope of the RF are markedly increased (see legend of color bar).

We used a sparse noise presentation paradigm to estimate the receptive fields of LFPs and multi-units (MUA). Sparse bar stimuli were flashed on a screen while the animal was rewarded for fixating a static red target (Figure 1A). The stimuli were located on a log-polar grid and scaled in length and width proportionally to eccentricity to account for the scaling of neuronal RFs with eccentricity (Motter, 2009).

We first removed the remnants of individual spikes on each electrode, using a Bayesian spike removal algorithm (Zanos et al., 2011b). We then determined whether the amplitude of the LFP or its power in different frequency bands were modulated by the stimulus. We filtered the signal in narrow frequency bands and applied the Hilbert transform to get an estimate of the instantaneous power of the signal as a function of time (Freeman, 2007). In this preliminary analysis, we estimated the receptive fields (RFs) of LFPs using reverse correlation (De Boer and Kuyper, 1968; Marmarelis and Marmarelis, 1978). We used these RFs to predict the signal in a separate validation dataset (see section “Methods” for details). We obtained poor predictions (mean *r* = 0.03 for array 1, *r* = 0 for array 2) for all examined frequency bands (Figures 1B,C, red lines). This is likely due to the short duration of each stimulus; generally, power modulations tend to be visible after sustained stimulation (Khawaja et al., 2009).

We repeated the analysis using the *amplitude* of the LFP in different frequency bands. This yielded considerably better predictions (Figures 1B,C, blue lines) across the 5 lowest frequency bands examined, encompassing the range from 0.5 to 30 Hz (mean *r* = 0.24 for array 1, *r* = 0.14 for array 2). Note that shaded error bars represent ±2 s.d.; the lack of overlap in the lowest 5 frequency bands indicates that the worst fits to the amplitude of the LFPs are nevertheless better than the best fits to the power of the LFP. Thus for the following analyses, we focused on the amplitude of the LFP filtered between 0.5 and 40 Hz.

### Receptive field profiles

Figure 1D shows a slice of a typical LFP RF, capturing the selectivity for space and orientation at a lag of 70 ms following stimulus onset. Casual inspection reveals little modulation of the spatial structure of the RF with orientation, aside from a scale factor. As this pattern of results was typical of both our LFP and MUA recordings, we assumed for the rest of the analyses that orientation tuning could be treated separately from space-time selectivity. As shown in Figure 1E, the consequent reduction in free parameters led to more reliable receptive field estimates (peak *z*-values shown next to color bars).

**Figure 2:**
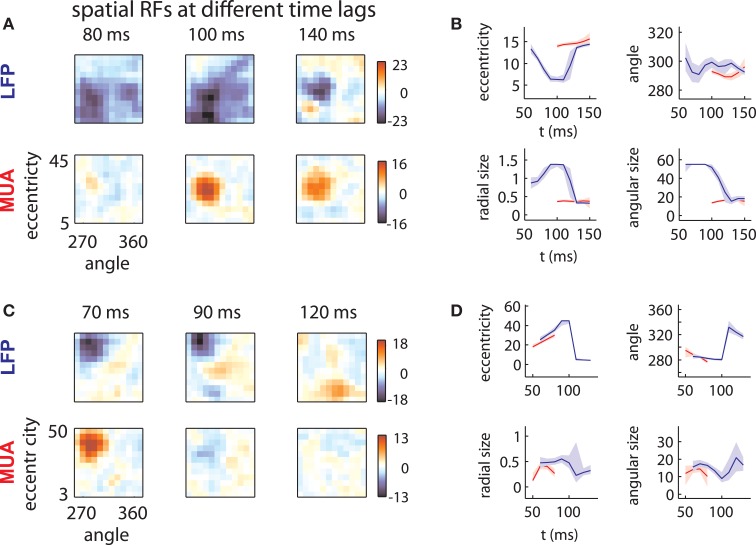
**LFP receptive fields change with time lag. (A)** Spatial envelope of an LFP RF (top) and MUA RF measured on the same electrode (bottom). The measured spatial envelope of the LFP RF (top) becomes markedly smaller at longer time lags, while the spatial envelope of the MUA RF (bottom) is stable. This pattern was typical for electrodes on the first array. **(B)** RF position and size as a function of time. LFP RF parameters (blue lines) corresponding to preferred eccentricity, polar angle, radial size, and angular size change markedly as a function of time. MUA RF parameters, illustrated in red, are comparatively stable. Parameters were estimated by fitting a Gaussian to the spatial RFs at different time lags and shaded error bars represent 95% confidence intervals for the parameters estimated through bootstrapping. **(C)** and **(D)**: as in **(A)** and **(B)** for an example electrode on the second array. Here, the LFP RF shows a late, foveal excitatory region (120 ms) far from the initial, peripheral preference (70 ms).

LFP spatial RFs were, however, strongly modulated as a function of time following stimulus onset. Figure 2A (top) illustrates the spatial RF of an LFP measured on one array at three different time lags. The LFP was responsive to a large area of the visual field at early time lags (80–100 ms) and a smaller area at later time lags (140 ms). This pattern of changes was typical of LFPs measured on this array, as shown below in more detail. By contrast, the MUA RF measured on the same electrode (Figure 2A, bottom) showed little evidence of such a change in size.

In addition to changes in size, LFP RFs frequently appeared to shift their preferred positions as a function of time. Figure 2C illustrates the receptive field of an LFP typical of the second array. Between 70 and 90 ms time lags, the RF shifted its preference toward high eccentricities. At 120 ms, the LFP responded to stimuli at a foveal location, far from the initial RF peak (peak *z*-value: 9.2; |z| = 4.5 corresponds to *p* = 0.001, corrected for multiple comparisons). Note that the polarity of the response reversed with time; while eccentric stimuli caused a low-latency negative deflection in the LFP signal, foveal stimuli caused a positive deflection at longer time lags. Again, the MUA RF (Figure 2C, bottom) showed no evidence of such a change.

To quantify these effects, we fit each RF time slice with a Gaussian, which captured the selectivity of the RF with four parameters: preferred eccentricity, preferred angle, radial size, and angular size. We estimated the uncertainty in these parameters through bootstrapping. Figures 2B,D show the changes in RF position (top) and size (bottom) for the example LFPs (blue) and MUAs (red). LFP RF parameter changes were highly significant across time lag (blue lines; shaded error bars correspond to 95% confidence intervals). By contrast, parameters for MUAs were comparatively stable across time lag (red lines), partly as a consequence of their shorter duration.

Hence, while the LFPs in the examples were well tuned for space, their spatial RFs were not static over time. As a result, the position and size of LFP receptive fields diverged substantially from those of corresponding MUAs at some time lags.

### Array analysis

**Figure 3:**
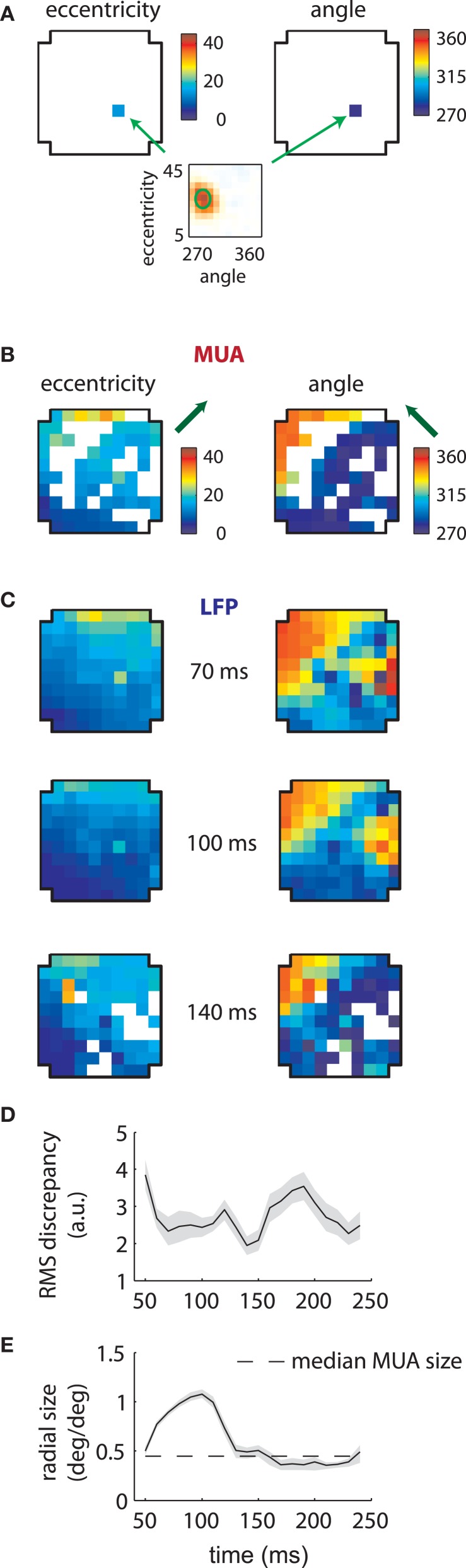
**MUA and LFP retinotopy—Array 1. (A)** Construction of retinotopies based on measured RFs. The preferred angle and eccentricity of a RF (bottom) is measured by fitting a Gaussian. These measurements are shown using a color code (top) at the location of the electrode on the array (cross-shaped outline). By repeating this process for all electrodes, the underlying retinotopy of the cortical sheet is revealed. **(B)** Measured retinotopy of MUAs. Electrodes yielding non-significant fits are left in white. The preferred eccentricity and polar angle change smoothly across the cortical sheet in a linear gradient (green arrows). **(C)** Measured retinotopy of LFPs as a function of time lag. The retinotopy evolves in a concerted fashion across time lag; at 140 ms, the array appears to represent more foveal locations and more locations around 270° polar angle. **(D)** Root mean square (RMS) discrepancy between MUA and LFP retinotopies as a function of time lag. MUA and LFP retinotopies are best matched at 140 ms time lag. Shaded error bars represent ±2 s.d. **(E)** Mean receptive field size as a function of time lag. The dashed line represent the mean MUA size measured on this array. The angular size of the RFs (not shown) showed a similar effect. Receptive fields are 3–4 times larger in linear dimensions at 100 ms compared to late time lags. Shaded error bars represent 95% confidence intervals for the mean.

Given the stability of MUA receptive fields across time lags (Figure 2), we refit the data on the assumption that their RFs were separable in time and space (see section “Methods”). We then plotted the estimated preferred eccentricity and polar angle of each MUA according to their location on the array. Figure 3A shows the result for a single MUA; here the RF was found at roughly 15° eccentricity along the vertical meridian in the lower visual field (bottom plot). These values were color-coded separately and displayed at the location of the recording electrode within the array (cross-shaped outlines). For eccentricity (left), blue colors corresponded to small values (foveal locations), while red corresponded to high values (eccentric locations). The same color similarly mapped the range of polar angle (right) from 270° (lower visual field) to 360° (right visual field), which spanned the ensemble of RFs we recovered for this array.

Repeating this process for every electrode revealed the retinotopy of MUAs across 4 × 4 mms of cortex (Figure 3B). Here eccentricity and angle for each electrode are coded as described above, with electrodes that did not yield significant RFs represented in white. As expected, preferred eccentricity and polar angle changed smoothly as a function of position on the array, and the direction of the eccentricity gradient, illustrated by a green arrow, was roughly orthogonal to that of polar angle.

LFP RFs exhibited a similar retinotopic organization, as illustrated in Figure 3C for the same array. As expected from the examples shown in the previous section, LFP retinotopy changed as a function of time lag. These changes were coherent across the array: at later time lags (140 ms), a greater proportion of the array responded to foveal locations (as shown by the increased representation of dark blue colors, left plot) and angles near 270° (again shown in dark blue, right plot). Figure 3D shows that the preferred angle and eccentricity of LFPs best matched those of MUAs at a time lag of 140 ms, as measured by the root-mean-squared (RMS) discrepancy.

As suggested by the example shown in Figure 2A, the mean LFP receptive field size changed dramatically as a function of time lag (Figure 3E). LFP RFs appeared larger at earlier lags, shrinking in size to a value close to that of the mean MUA receptive field size at longer time lags (dashed line).

**Figure 4:**
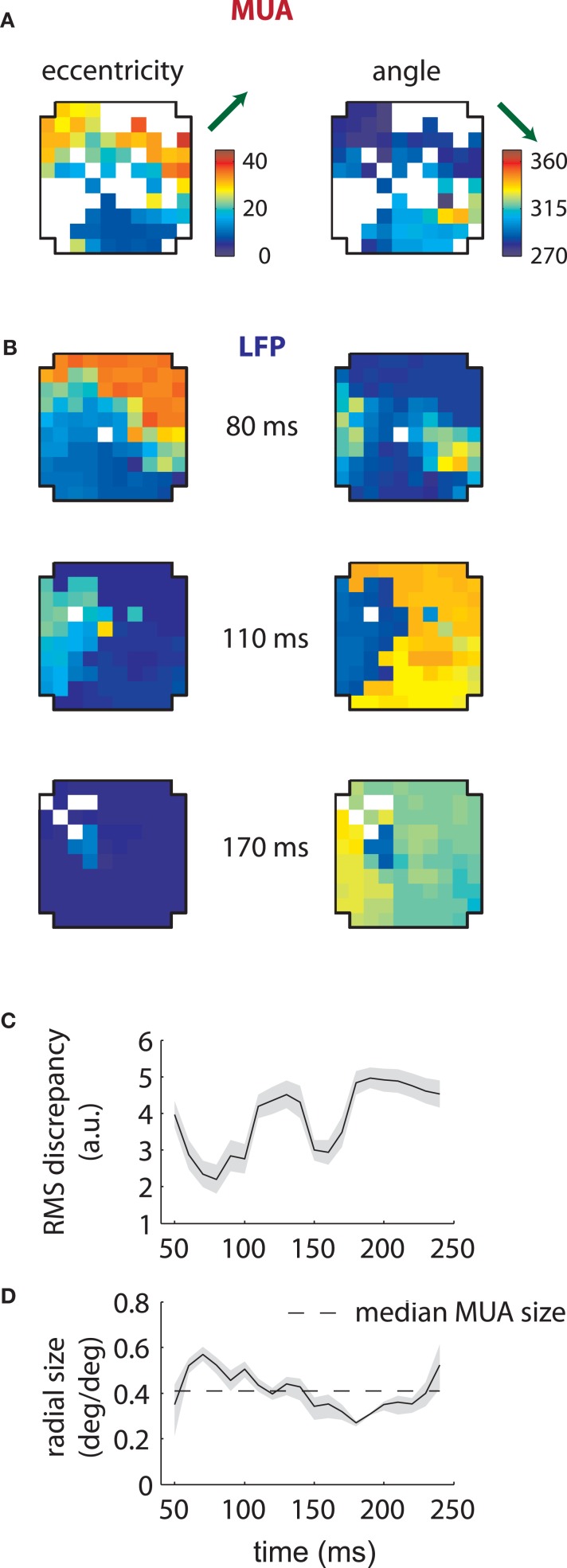
**MUA and LFP retinotopy—Array 2. (A–D)** as in Figures 3(B–E), now for the second array. The second array shows qualitatively different changes in retinotopy as a function of time. In particular, while at early time lags (80 ms) the array forms a smooth retinotopy, at later time lags (170 ms) the entire array represents a foveal location.

Corresponding results for the second array are shown in Figure 4. In this case LFP RFs formed a retinotopic map at early time lags (Figure 4B, top), consistent with that of MUAs, with the best match occurring at 80 ms (Figure 4C). Strikingly, however, a foveal component appeared at later time lags, overtaking the retinotopy of the LFPs completely by 170 ms (Figure 4B, bottom). Thus, the majority of LFPs measured in the second array showed biphasic receptive fields similar to that illustrated in Figure 2C. The changes in retinotopy were accompanied by modest changes in mean RF size (Figure 4D).

These results show that the LFP retinotopy changes with time lag in a concerted fashion across the cortical surface. While at some time lags the LFP retinotopy matched that of the MUAs, at others it considerably diverged. Thus, LFP RFs reflect more than the underlying retinotopy of MUAs.

Furthermore, the relationship between LFPs and MUAs was qualitatively different between the two arrays. For the first array, the retinotopy of the LFPs best matched those of MUAs at late time lags (140 ms); in the second array, LFP RFs were aligned with MUAs at early time lags (80 ms). In addition, our second array showed an array-wide foveal component unseen in the first array.

### Robustness of retinotopy

The striking differences between MUA and LFP receptive fields within an array and in the results between arrays could conceivably be a signature of a transient electrical artifact; that is, a source of noise that contaminated recordings on a particular recording day, such as line noise, reward artifact, cross-talk between electrodes, etc. To examine this, we repeated the analyses for data recorded on another day in each array. LFP RFs estimated on other recording days exhibited the same qualitative pattern of shift in retinotopy across time lag characteristic of each array. The day 1 LFP-day 2 LFP RMS discrepancy, averaged across time lags, was 0.8 for both arrays. By contrast, at the optimal temporal lag, the within-day LFP-MUA RMS discrepancy was ~2 (Figures 3D, 4C). The tuning of the LFP is thus consistent across days.

It is also possible that signal processing exaggerated the differences between the two signals. The results presented in Figures 3B and 4A reflect MUA obtained by full-wave *rectifying* a band-pass filtered (750–3500 Hz) signal; we refer to this as the rMUA (cf. Xing et al., 2009). The MUA is also commonly defined by the density of *threshold* crossings in band-pass filtered voltage traces (Katzner et al., 2009); we term this the tMUA. These different definitions could potentially isolate different components of the signal (Supèr and Roelfsema, 2005). We thus repeated our analyses for the tMUA (see section “Methods” for details).

We found similar retinotopies with both measures of multiunit activity, with rMUA-tMUA RMS discrepancies of 0.4 and 0.9 for arrays 1 and 2, respectively. We found fewer significantly tuned electrodes with the threshold method, however, especially in the second array (tMUA: *N* = 57 in array 1, *N* = 23 in array 2; rMUA: *N* = 65 in array 1, *N* = 69 in array 2). We found that tMUA RFs were slightly (7%), but significantly smaller than rMUA receptive fields (*p* < 0.05 for each array, two-sided Wilcoxon rank sum test). These results are consistent with the rMUA having similar properties to the tMUA, while integrating over a slightly larger cortical area.

Thus, the changing retinotopy across time lags and the qualitatively dissimilar properties of the MUA and LFPs are unlikely due to transient noise sources or to the choice of data preprocessing for the MUA (above) or the LFP (Figures 1B,C).

### Temporal mixture model

The previous section showed that at some time lags, LFP RFs are organized in a retinotopic fashion similar to MUAs. Yet, this close correspondence between LFP and MUA retinotopy is broken at other time points. In the second array, in particular, the appearance of a foveal component at late time points (Figure 4B) hints at the interplay between an MUA-like retinotopic component, which changes from electrode to electrode, and a component tuned for foveal locations, shared by all electrodes.

**Figure 5:**
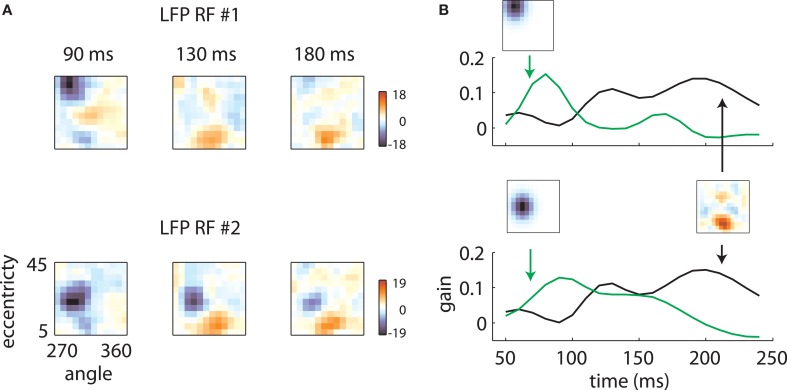
**Temporal mixture model. (A)** Two LFP RFs measured on the same array are illustrated. At early time lags, the RFs are quite different, with the first RF representing high eccentricity locations and the second representing intermediate eccentricities. At late time lags, however, both RFs show a secondary excitatory lobe in a foveal location. **(B)** These results are explained by positing that each RF is a mixture of two components: a retinotopic component, specific to each electrode, constrained to take the shape of a Gaussian, and a shared component, which can take an arbitrary shape but is shared across electrodes. Each RF time slice is obtained by a weighted sum of the electrode-specific retinotopic component (green lines) and the shared component (black line). The relative strength of the two components as a function of time determines the shape of the RFs.

Figure 5 illustrates how the interplay between these two mechanisms might account for the data. LFP RF time slices measured on two electrodes on the same array are plotted in Figure 5A. While at early time lags (left and center), the RFs are markedly different, they have similar shapes at later time points (right). These results are explained in Figure 5B by the interplay of electrode-specific components (green lines) and a second component (black lines) shared by all electrodes on the same array. Here the changing receptive field positions result from electrode-specific response components that are stronger at early time lags and a shared component that is stronger at later time lags.

More formally, we assumed that the RFs *f*^*e*^_τ, *r*, θ, *o*_ as a function of electrode number *e*, time lag τ, eccentricity *r*, angle θ and orientation *o* were given by:

(1) $$f_{\tau ,\;r,\;\theta ,\;o}^{e}=a_{\tau }^{e}\;p_{r,\;\theta ,\;o}+b_{\tau }^{e}q_{r,\;\theta ,\;o}^{e}$$

Here *p*_*r*, θ, *o*_ is a component shared by all electrodes on a given array and *q*^*e*^_*r*, θ, *o*_ is specific to each electrode; they are weighted differentially depending on electrode number and time lag by factors *a*^*e*^_τ_ and *b*^*e*^_τ_. The shared component *p*_*r*, θ, *o*_ could take on any spatial configuration. The electrode-specific component *q*^*e*^_*r*, θ, *o*_ was constrained to have a Gaussian spatial RF profile and a separable orientation tuning curve.

We fit the temporal mixture model for each array by minimizing the squared error between the model and the data (see section “Methods” for details). The resulting model yielded a highly significant improvement over a baseline model without the constant component (0.57 vs. 0.45 R^2^, *p* « 0.001 for array 1; 0.37 vs. 0.30 R^2^, *p* « 0.001 for array 2; *F*-test).

**Figure 6:**
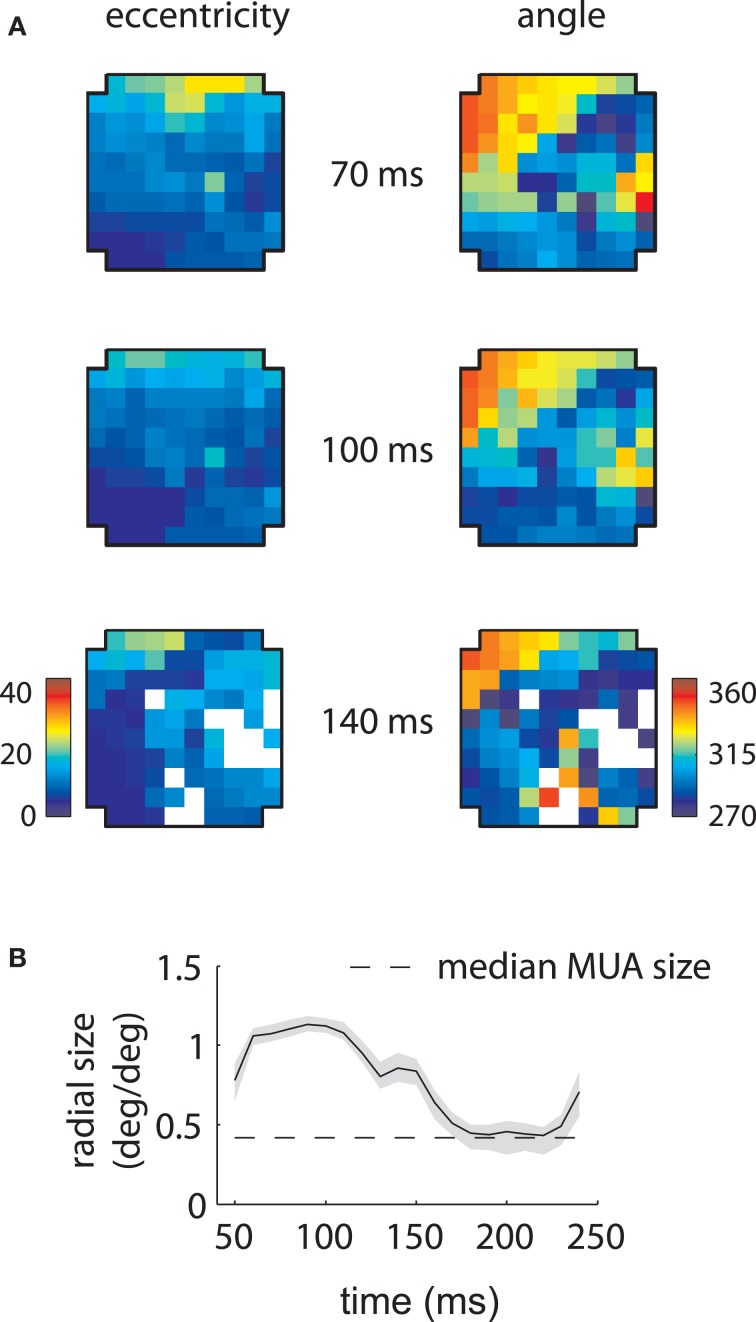
**Reconstructed retinotopies based on temporal mixture model—Array 1. (A)** Reconstructed retinotopy of the array as a function of time. These retinotopic maps were obtained by fitting the temporal mixture model to the array 1 data, creating simulated RFs based on the measured parameters, and fitting the simulated RFs with Gaussians. The mixture model captures the greater representation of low eccentricity and 270° locations at late time lags (140 ms). **(B)** Reconstructed mean RF size as a function of time. The model captures the dramatic change in measured RF size as a function of time. Shaded error bars represent 95% confidence intervals for the mean.

Based on the model fits, we reconstructed composite LFP RFs that were then fit with Gaussians at every time point to reconstruct retinotopies. These are illustrated in Figure 6 for array 1. The simulations replicated the pattern of increased representation of low eccentricity and 270° locations at longer time lags (Figure 6A) and the apparent decrease in receptive field size in time (Figure 6B).

**Figure 7:**
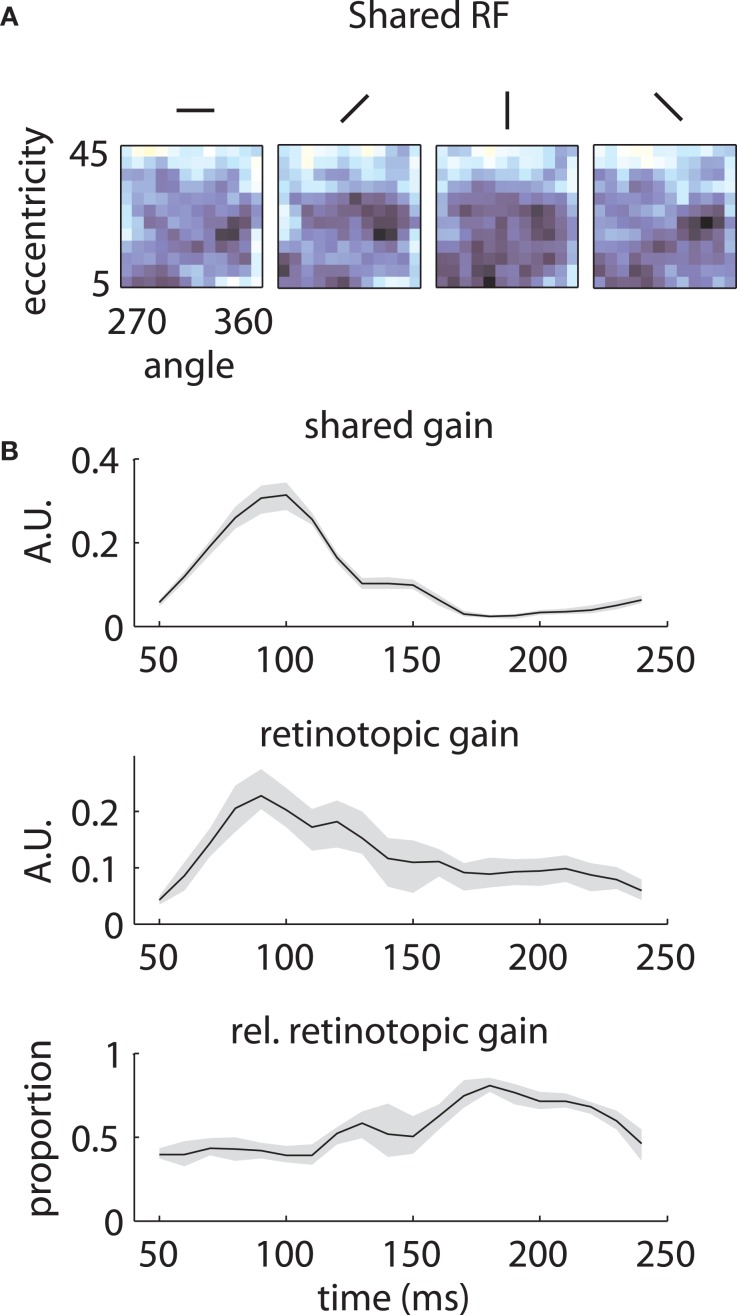
**Temporal mixture model parameters—Array 1. (A)** Shared receptive field estimated from the data. The RF is broadly tuned for space and orientation. **(B)** Gains of each component as a function of time. Both the median shared gain (top) and the retinotopic gain (middle) peak at early time lags. However, the retinotopic gain decays more slowly as a function of time. Therefore, the retinotopic gain is relatively larger at late time lags (bottom). This creates a shift in the representation from broadly tuned (shared component) to more tightly tuned (retinotopic component). Shaded error bars represent 95% confidence intervals for the median.

The underlying mechanism for this switch is illustrated in Figure 7. The shared component was triggered by stimuli of any orientation across a fairly broad region of space (Figure 7A), with peak selectivity at central locations (~10° eccentricity, 315° polar angle). In absolute terms, both the shared and retinotopic components were strongest at early time lags (Figure 7B). However, because the retinotopic component decayed more slowly, it was relatively stronger at late time points (Figure 7B, bottom). It follows that at early lags, the observed RFs were both more broadly spatially tuned and biased toward representing central locations than at later lags.

**Figure 8:**
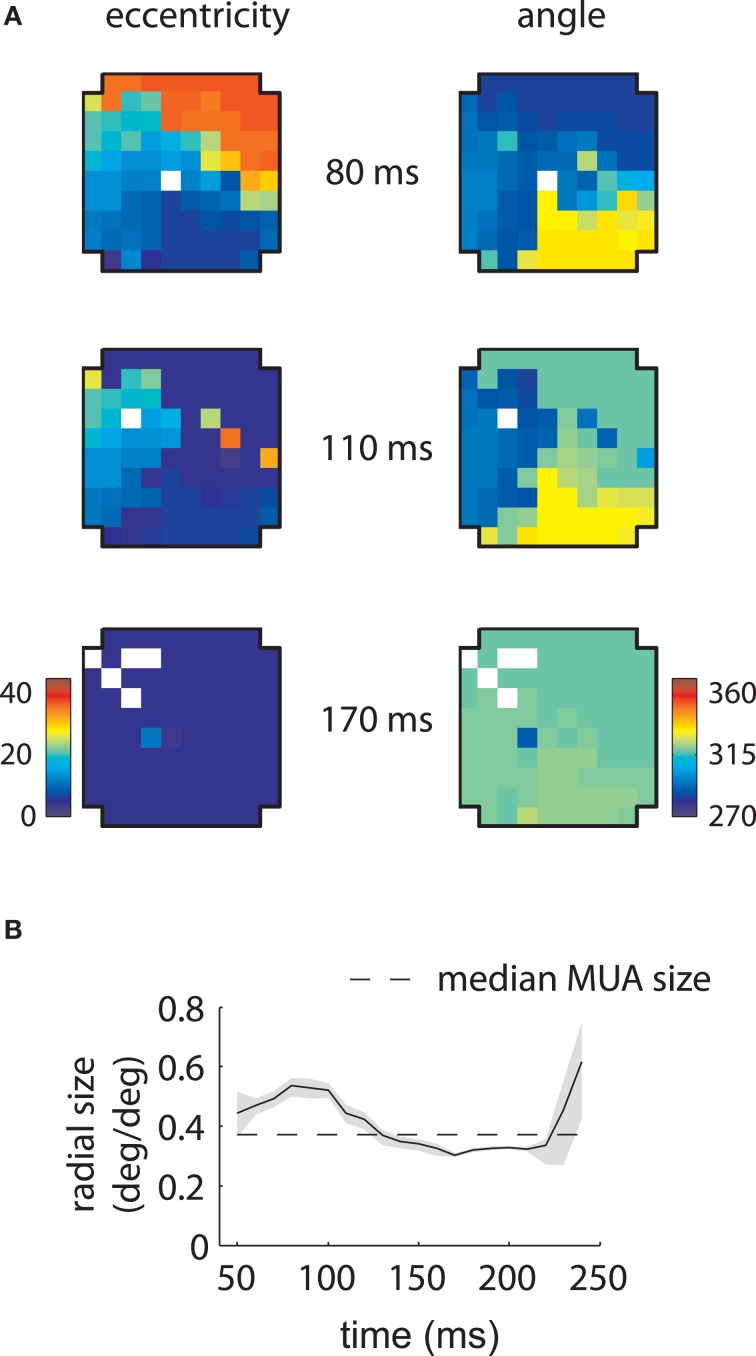
**Reconstructed retinotopies based on temporal mixture model—Array 2. (A)** and **(B)** as in Figure 6. The model captures the change in the representation from retinotopic at early time lags to exclusively representing foveal locations at late time lags.

**Figure 9:**
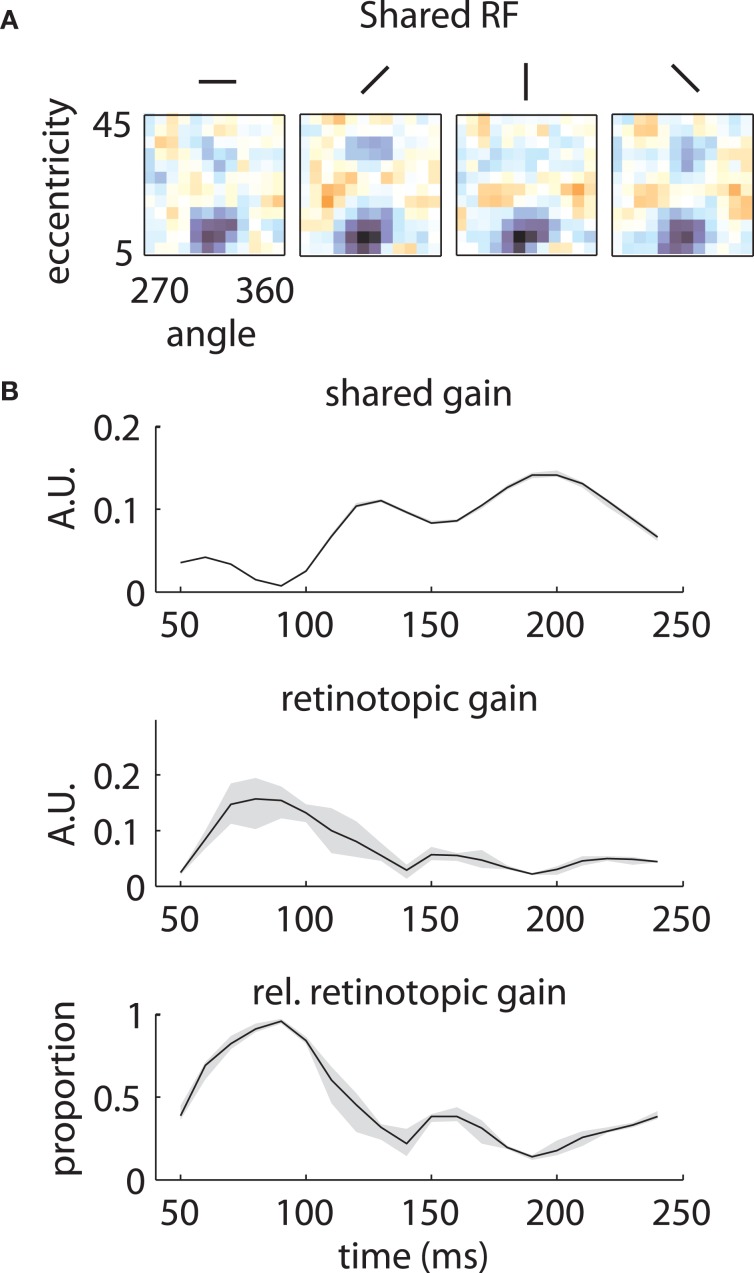
**Temporal mixture model parameters—Array 2. (A)** and **(B)** as in Figure 7. The shared RF is tightly tuned for foveal locations. The gain of the shared RF grows larger at late time lags. Therefore, the RFs switch from an early retinotopic to a purely foveal late representation.

Similar results are shown for the second array in Figure 8. The model captured the gradual overtaking of the array by a constant component at later time lags (Figure 8A), along with the decrease in receptive field size with increasing lag (Figure 8B). Figure 9A shows that this shared component was strongly tuned for a foveal portion of the visual field. As with the first array, the retinotopic component peaked at early time lags (Figure 9B, middle); unlike the other array, however, the shared component manifested itself mostly at later time lags (Figure 9B, top).

Together, these results explain the observed changes in retinotopic organization in terms of a gradual switch in the importance of two distinctly tuned components. The first, retinotopic component was strongest in both arrays at early time lags, while the second, shared component differed qualitatively between the two arrays. Thus, shared components may represent idiosyncratic, large-scale biases in visual representation (Jia et al., 2011), a matter we explore in more detail in the discussion.

### Retinotopic component

**Figure 10:**
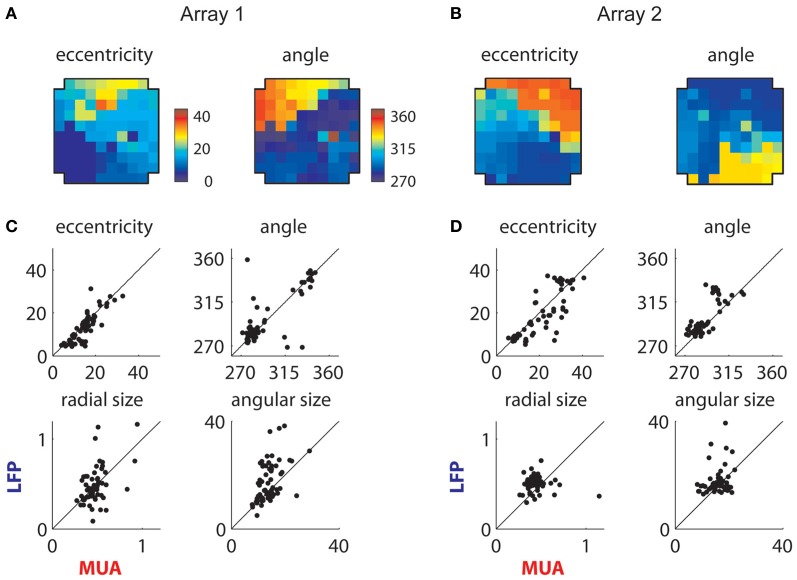
**Retinotopic components. (A)** Measured retinotopy of retinotopic component for Array 1 and **(B)** for Array 2. These can be compared to the corresponding MUA-based estimates in Figures 3B and 4A. **(C)** Receptive field parameters of LFPs and MUAs measured on the same electrode compared for Array 1 and **(D)** Array 2. Eccentricity and angle match between MUAs and LFPs, while LFPs display larger receptive fields on average.

The extracted retinotopic components are illustrated in Figures 10A,B; they were retinotopically arranged in a manner similar to MUAs (Figures 3B and 4A). This link is shown in more detail in Figures 10C,D, which compares the positions (top row) and sizes (bottom row) of LFP and MUA RFs measured on the same electrodes. The eccentricity and polar angle of the extracted RFs were similar to those of MUAs measured at the same location, save for a cluster of observations at the bottom right of the second array (Figure 10B). These electrodes had a retinotopic component that was foveal and thus overlapped with the shared component; this made precise estimation of their location and size problematic.

Consistent with previous literature, retinotopic LFP RFs were larger than corresponding MUA receptive fields (Figures 10C,D); mean and median sizes are documented in Table 1. We estimated the integration radius of the LFP using the method introduced in (Xing et al., 2009). This involves first estimating the integration radius in visual coordinates, then translating this into cortical coordinates σ_cLFP_ using the estimated cortical magnification factor (*m*), according to the formula:

(2) $$\sigma_{c\text{LFP}}={\left[m^{2}(\sigma_{v\text{LFP}}^{2}-\sigma_{v\text{MUA}}^{2})+\sigma_{c\text{MUA}}^{2}\right]}^{1/2}$$

Here σ_vLFP_ and σ_vMUA_ correspond to the mean size of LFP and MUA RFs in visual coordinates, respectively, and σ_cMUA_ is the integration radius of the MUA in cortical coordinates. Because the retinotopy of V4 is less regular than in V1, magnification can change depending on the position on the cortical surface. We therefore estimated *m* by averaging the magnitude of the gradients of eccentricity and polar angle across the array (see section “Methods” for details). This yielded an integration radius of 300 microns [95% CI: (100,500)] for the retinotopic component of the LFP in array 1 and 400 microns [95% CI: (150,650)] in array 2. Thus, the retinotopic component of the V4 LFP arises from the integration of activity proximal to the electrode, consistent with previous results in V1 (Katzner et al., 2009; Xing et al., 2009).

**Table 1:** **Summary statistics of measured RF sizes (in parentheses: 95% confidence intervals estimated through bootstrapping)**.

	**Radial size—Median**	**Radial size—Mean**	**Angular size—Median**	**Angular size—Mean**
LFP	0.50 (0.50, 0.51)	0.54 (0.51, 0.57)	16.6 (16.2, 16.8)	18.0 (17.2, 19.0)
MUA	0.44 (0.42, 0.45)	0.46 (0.44, 0.48)	14.0 (14.4, 15.7)	15.0 (14.4, 15.7)

### Orientation and temporal tuning

Additional information about the relationship between the MUA and the LFP may be gained by comparing the orientation and temporal tuning of the two signals. We found that the LFPs were essentially untuned for orientation, with a mean circular variance (CV; Ringach et al., 2002) of 0.95 for the retinotopic component (minimum CV: 0.88) and 0.94 for the shared component across both arrays. On the other hand, some MUAs were tuned for orientation (min CV: 0.65), with a mean CV of 0.87 for significantly tuned MUAs across both arrays. This data is consistent with the idea that the LFP integrates over a larger area than the MUA, although the poor tuning prevents further analysis of the integration radius in the manner of Katzner et al. (2009).

**Figure 11:**
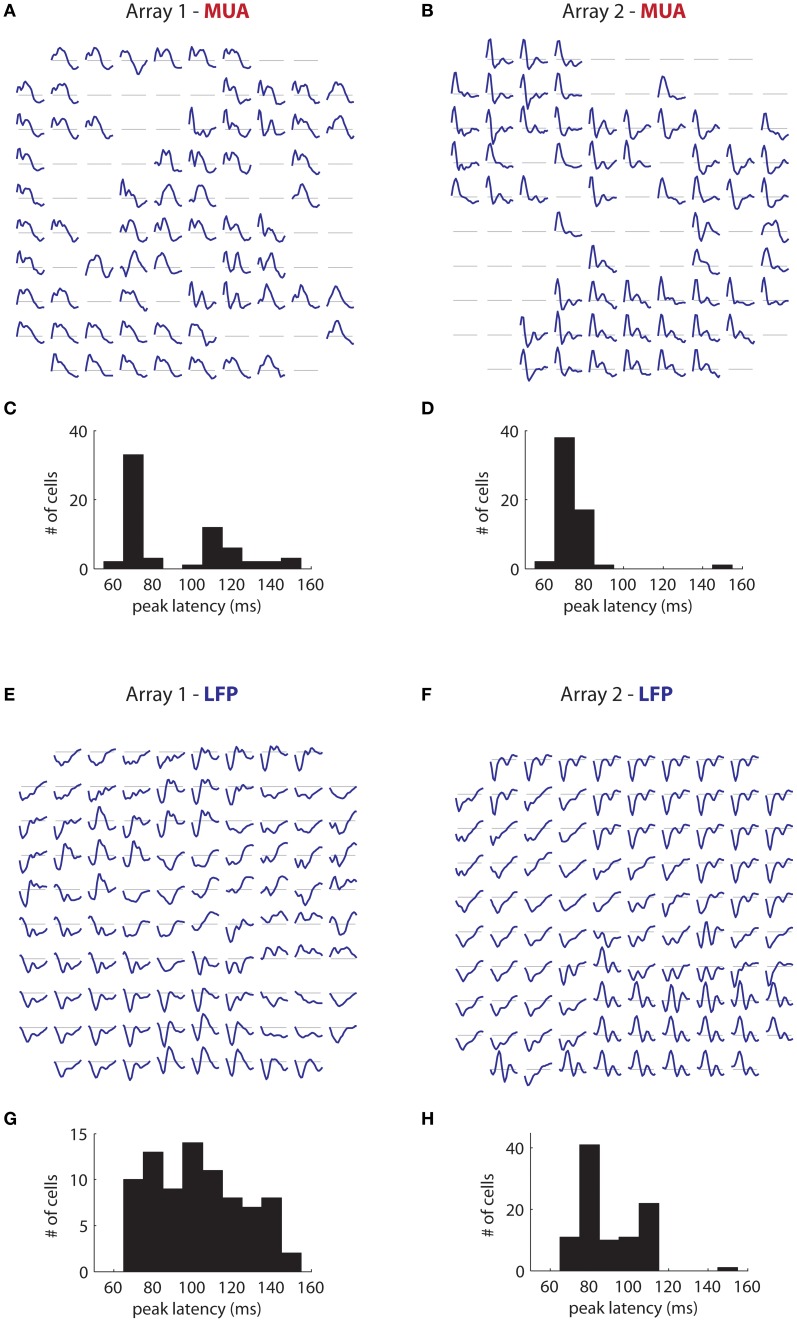
**Temporal filters. (A)** Estimated temporal filters for the MUAs measured on the first array and **(B)** On the second array. Filters span 50–240 ms time lags. Gray lines correspond to a coefficient of 0. Filters are highly stereotyped across each array. **(C)** Distribution of peak latency for the MUAs for the first array and **(D)** The second array. Peak latency is centered around 70 ms. **(E,F)** Same as in **(A)** and **(B)**, but for the retinotopic component of the LFPs. LFP temporal filters vary much more than corresponding MUA temporal filters, Their duration is generally longer, and they are smoother. **(G)** and **(H)** LFP peak latencies. These are more widely distributed than corresponding MUA peak latencies, and generally longer.

More interesting is the temporal tuning of both signals. Figure 11A illustrates the temporal filters of significantly tuned MUAs (blue lines; 50–240 ms) as a function of their position on the first array. Temporal filters are stereotyped through the array, with a rapid rise followed by a slower decay. There is some indication of suppression at late time lags (segments below the gray line). The majority of filters have a peak latency of 70 ms (Figure 11C), with a minority having a peak around 120 ms. These results are mirrored in array 2 (Figure 11B), where the filters are also highly stereotyped, although here the decay appears faster. The peak latency is also centered around 70 ms (mean: 73 ms; Figure 11D).

These results contrast strongly with the time filters of the retinotopic component of the LFP in array 1 (Figure 11E), in which the temporal filters differed dramatically across the array. Moreover, the filters are of considerably longer duration than the corresponding MUA filters, reflecting the fact that the signal is modulated at low frequencies (Figure 1B). While the dominant polarity is negative, some filters have roughly equal positive and negative polarity phases (bottom center) or have mostly positive polarity (right middle). The peak latency occurs late compared to the MUA, and varies widely (mean: 118 ms; Figure 11G). Similar trends are visible for array 2 (Figure 11F), with filters showing large variation in shape and polarity. Peak latency is also more broadly distributed than the corresponding MUA data, and longer, with a mean of 90 ms (Figure 11H).

Thus, while MUAs and the retinotopic component of LFPs have similar retinotopy (Figure 10), the two signals diverge strongly in terms of temporal selectivity. LFPs have more sluggish dynamics, longer duration, and longer peak latencies; they are also more variable in shape and polarity than MUAs. One must therefore be careful in interpreting the LFP as a spatially smoothed version of the MUA, since the LFP does not reflect the MUA per say, but rather subthreshold activity (Buzsáki et al., 2012). While in some instances, like retinotopy, the relationship between subthreshold activity and the MUA is sufficiently well understood to make the relationship between LFP and MUA transparent (Carandini and Ferster, 2000), in the case of temporal tuning the relationship is complex, and the LFP and MUA reveal themselves as highly distinct.

## Discussion

### General discussion

The local field potential is a complex signal which offers a window into cortical processing at larger spatial and longer temporal scales than those associated with single units. Components of this signal have been shown to correlate with attention (Fries et al., 2001, 2008; Gregoriou et al., 2009), cortical inhibition (Henrie and Shapley, 2005; Atallah and Scanziani, 2009), arousal (Van Swinderen et al., 2004; Andretic et al., 2005), synchronicity (Gray and Singer, 1989; Mukamel et al., 2005; Nir et al., 2007), and other network phenomena.

While the LFP has proven a highly interesting signal, its interpretation has been marred by our lack of understanding of its biophysical sources and its relationship to spikes. Action potential generation and passive propagation have been well understood for several years (Hodgkin and Huxley, 1952; Koch, 1999). By contrast, LFPs have only recently been modeled in a biophysically detailed fashion (Bedard et al., 2006; Milstein et al., 2009; Lindén et al., 2011; Buzsáki et al., 2012). Modeling studies have unequivocally concluded that the LFP is an intrinsically more complex signal than spikes, reflecting a variety of distinct electrical phenomena (Buzsáki et al., 2012). Lindén et al. (2011) show that the integration radius of the LFP depends both on cortical layer and the correlation structure of the input.

It follows that the structure of the LFP and its relationship to spikes may well vary from area to area in idiosyncratic and unpredictable ways. We thus set out to estimate the receptive fields of LFPs in area V4 of two macaques and compared their properties to those of MUAs. Our results show that, in the context of a sparse noise presentation paradigm (reverse correlation), where the LFP signal is dominated by transient as opposed to sustained activity, the LFP reflects multiple sources of inputs.

In both our subjects, one component of the LFP reflected the underlying retinotopic organization of the cortical sheet (Figure 10). This component was strongest at 80–90 ms following stimulus onset (Figures 7B and 9B), and decayed slowly to baseline at 200–250 ms. It arose from the integration of activity within a patch of cortex of ~350 um. Retinotopic signals were mixed with components shared by all electrodes on a given array. In the case of our first array, the shared component was broadly tuned for space and peaked at early time lags (Figure 7). For the second array, the shared component was tuned for foveal locations and peaked at late time lags (Figure 9).

What is the source of the shared component? We used state-of-the-art signal processing to eliminate potential signal distortion by analog filters and spike remnants (Nelson et al., 2008; Zanos et al., 2011b; see section “Methods” for details). While it remains possible that the shared component is artifactual, its tuning properties are inconsistent with distortion caused by faulty grounding, for example. In the second array, in particular, we see that the shared component has temporal tuning properties which are very different from MUAs or early LFPs recorded on the array. Careful inspection of the shared component measured at late time lags (Figure 4B, 170 ms) shows that there is a small but visible gradient in angular selectivity from the left to the right of the array; this gradient is not captured by the temporal mixture model (Figure 8A).

Hence, the shared components actually change across the array, albeit more modestly than the retinotopic components. We thus hypothesize that the shared components reflect large-scale biases in the input to area V4. Jia et al. (2011) found that one component of low-gamma LFPs in V1 have similar orientation tuning across 4 mm of cortex, independent of the preference of local MUAs. It has been hypothesized that this reflects large scale biases in orientation representation in striate cortex, where orientations aligned with the preferred polar angle of neurons are slightly overrepresented (Freeman et al., 2011). Such large-scale biases in representation, which have no functional role per say, could vary idiosyncratically from animal to animal. We conjecture that this could explain the sharp difference between the arrays in the tuning of the shared common component.

Another potential source of discrepancy in the tuning of the shared component lies in the sampling of cortical layers. Different layers could be targeted as a function of position due to the curvature of cortex. The changing polarity of the temporal filters in Figures 11E,F is consistent with this idea. The second animal was much smaller (5–6 kg) than the first animal (9–10 kg). Consequently, the distance between the lunate and superior temporal sulci at the level of the implant was smaller in the second animal (4–5 mm vs. 6–7 mm) and the cortical surface was more curved. Thus, it is unlikely that the sample of layers is exactly the same in both animals, although this could not be verified histologically. These factors highlight that as an epiphenomenal signal (although see Anastassiou et al., 2011), the LFP is complex and noisy, and care must be taken in its interpretation.

### The integration radius of the LFP

These results may bear on the continuing debate regarding the integration radius of the LFP. We and others have demonstrated that different components of the LFP have tuning properties which are consistent with either local (~300 um) or global (several mms) integration of MUA activity (Kreiman et al., 2006; Liu and Newsome, 2006; Katzner et al., 2009; Xing et al., 2009; Jia et al., 2011). We thus conclude that it is not meaningful to speak of *the* integration radius of the LFP, for the simple reason that the LFP reflects *multiple* sources of inputs with different integration scales.

We conjecture that the discrepancies in previous studies are due to signal processing and analysis choices which enhance the relative strength of one component of the LFP over another. An important distinction is that some studies (Katzner et al., 2009; Xing et al., 2009) have examined the amplitude of the LFP, similar to what is done here, while others (Kreiman et al., 2006; Liu and Newsome, 2006; Jia et al., 2011) have examined power at a selected frequency. The amplitude of the LFP is sensitive to transients in the local field potential, while power is sensitive to sustained oscillatory activity. Lashgari et al. (2012) have found that transient and sustained LFPs in V1 have markedly different tuning properties; this translates into changing relationships with MUA activity. It will be interesting to compare the retinotopy of amplitude and power components of the LFP directly in a paradigm which triggers both stereotyped deflections and oscillatory activity; natural movies may be able to evoke both phase and power modulations (cf. Figure 6, Rasch et al., 2008).

Another potential source of variability in the properties of the LFP may result from the treatment of the dimension of time. Katzner et al. (2009), for instance, analyzed the first component of the Singular Value Decomposition of the temporal-orientation tuning curve. Xing et al. (2009) instead analyzed responses at a latency corresponding to the peak deviation of the LFP. Such choices would not permit analysis of the multi-component temporal responses of the kind we have reported here (Figure 2).

It may well be the case that in V1 the major contribution to the LFP is separable with respect to time lag and highly local. In this respect, V1 may be a special case, as its retinotopy is remarkably precise (Hubel and Wiesel, 1977; Blasdel and Fitzpatrick, 1984; Ohki et al., 2005, 2006); by contrast, higher-level areas have less precise retinotopy. Tonotopy in primary auditory cortex is significantly less precise than retinotopy in V1, at least in rodents (Castro and Kandler, 2010). Interestingly, LFP spectrotremporal receptive fields are much more broadly tuned than those of MUAs in both cat (Eggermont et al., 2011) and monkey (Kajikawa and Schroeder, 2011).

Given that the integration radius of the LFP varies with the correlation structure of the input (Lindén et al., 2011), part of the disagreement may reflect genuine inter-areal differences in the LFP. While this complicates the interpretation of the LFP, it may afford an opportunity to study how input correlation structures are forwarded and modified in a hierarchy, with wide implications for our understanding of encoding and decoding neural activity (Averbeck et al., 2006).

## Methods

### Task

The recording methods have been described in detail previously (Zanos et al., 2011a). Briefly, we implanted chronic microelectrode *Utah* arrays in area V4 of two macaques (*Macaca mulatta*). Area V4 was identified based on stereotactic coordinates and anatomical landmarks (Ghose and Ts'O, 1997). After recovery, the monkey was seated comfortably in a primate chair (Crist Instruments) and trained to fixate for liquid reward. Eye position was monitored at 200 Hz with an infrared camera (SR Research). All aspects of the experiments were approved by the Animal Care Committee of the Montreal Neurological Institute and were conducted in compliance with regulations established by the Canadian Council of Animal Care.

### Signal acquisition and processing

We recorded wideband signals at 10 kHz (bandpass filtered in hardware between 0.07 and 2500 Hz) over the 96 channels of each Utah array. We monitored the power spectrum of recorded wideband signals on a daily basis to minimize line noise and other artifacts. Recordings were referenced against a ground located on the skull 2–3 cm away from the array. The same recording equipment was kept in place for both animals.

The wideband signal was band-pass filtered between 750 and 3500 Hz, rectified, band-passed between 2 and 40 Hz, and downsampled to 100 Hz to form the MUA signal (Xing et al., 2009). For comparison with previous literature, we also computed an alternative MUA based on applying a low threshold (3σ) to the wideband signal bandpassed between 750 and 3500 Hz (Katzner et al., 2009). We used a detection deadtime of 1 ms, a two-sided threshold, and binned the events at 100 Hz (10 ms time bins); this gave similar results to the rectification-based method (see section “Results,” Robustness of retinotopy). Action potential artifacts were removed from the wideband signal using a Bayesian method (Zanos et al., 2011b); the despiked wideband signal was then downsampled and band-pass filtered to produce the LFP (see Preliminary Analysis and Receptive Field Estimation section for filters specific to each analysis).

### Stimulus

Each animal was trained to fixate a red spot (2° fixation window) while sparse dark bar stimuli were flashed on a uniform gray screen. The monitor was refreshed at a rate of 75 Hz; stimuli changed every odd frame (37.5 Hz); and each bar stimulus lasted for 6 monitor frames (12.5 Hz; 80 ms). Stimuli were presented in a single continuous trial, which lasted 25 min for the data presented for the first array and 30 min for the data presented for the second array. We repeated the experiment on other recording days for each array, with similar results (data not shown). The bar stimuli were placed along a 12 × 12 polar grid (Figure 1A), such that stimuli at the periphery were longer and wider than those near the fovea; bars scaled linearly with eccentricity. The length of the bars was chosen so that no bars touched when presented simultaneously; bar width was set to 0.25 times the eccentricity. Four different orientations were used. The grid was confined to the lower right corner of the screen. It spanned 120° of polar angle and 5–40° of eccentricity for array 1 and 3–50° of eccentricity for array 2. On average, 7 bar stimuli were on the screen at any given time.

### Preliminary analysis

In a preliminary analysis (Figures 1B,C), we first downsampled the despiked wideband signal to 200 Hz, then filtered it in seven bands (Freeman, 2007): delta (0.5–4 Hz), theta (4–8 Hz), alpha (8–12 Hz), low beta (12–20 Hz), high beta (20–30 Hz), low gamma (30–50 Hz), and high gamma (50–80 Hz). We also took the absolute value of the Hilbert transform of each band-passed filtered LFP to obtain estimates of the instantaneous power in each frequency band.

We split the data into a fit dataset and a validation dataset: for each 5 s block of the data, the first 4 s were assigned to the fit dataset and the last second to the validation dataset. We estimated orientation-spatial-temporal receptive fields for both band-pass filtered LFPs and their power using standard reverse correlation on the fit dataset (De Boer and Kuyper, 1968; Marmarelis and Marmarelis, 1978; Figures 1B–E). We applied a Gaussian spatial kernel (σ = 0.8) to the estimated receptive fields and predicted the signal in the validation dataset, assuming a linear model with the estimated filter.

### Receptive field estimation

The results of the preliminary analysis (previous section, Figures 1B,C) showed that the stimulus modulated the amplitude of the LFP but not its power, and that the modulation was concentrated at low frequencies. For the remaining analyses, we thus band-pass filtered the despiked wideband signal in the range (0.5–40 Hz) and downsampled to 100 Hz to produce the LFP signal. Inspection of the reverse correlation filters led us to a low-dimensional parameterization for each temporal slice of the LFP receptive fields: the selectivity of the RF is given by the product of an orientation filter and a Gaussian spatial envelope. Specifically, we assumed that the contribution of the stimulus presented τ epochs ago to the internal response in the *k*th time bin was given by:

(3) $$\eta_{k}=\sum_{r,\;\theta ,\;o}s_{k-\tau ,\;r,\;\theta ,\;o}v_{o}G_{r}(r_{0},\;\sigma_{r})G_{\theta }(\theta_{0},\;\sigma_{\theta })+d$$

*s*_*k* − τ, *r*, θ, *o*_ is the stimulus presented τ epochs ago. *d* is a bias. *G*_*a*_(*b*, *c*) is a Gaussian curve evaluated at *a*, centered around *b*, with width (standard deviation) *c*. *v*_*o*_ is the orientation filter. We fit the model for each time slice τ = 1 to 24 by least-squares. We initialized the parameters by fitting a Gaussian to the spatial envelope of the reverse correlation estimate of the filter through least-squares; this envelope was determined by taking the first singular vector of the SVD of the reverse correlation estimate (Ahrens et al., 2008).

The model for MUAs was similar, but this time we assumed that the receptive fields did not change in shape across time slices, but were simply scaled by a time-dependent gain:

(4) $$\eta_{k}=\sum_{\tau ,\;r,\;\theta ,\;o}s_{k-\tau ,\;r,\;\theta ,\;o}u_{\tau }v_{o}G_{r}(r_{0},\;\sigma_{r})G_{\theta }(\theta_{0},\;\sigma_{\theta })+d$$

Here *u* corresponds to the weights of a separable time filter. We fit the model through least-squares.

An MUA RF was deemed significantly tuned if the fit was significant at the *p* < 0.0001 level according to a χ^2^ test (Wood, 2006). We found this criterion too lenient for LFP RFs, presumably because the correlation structure of the LFP did not follow the assumptions of the test. Instead, an LFP RF fit was deemed significant if its *R*^2^ value was greater than the observed *R*^2^ values on any electrode on the same array for time slices from 10 ms to 40 ms. Importantly, this simulation-based method preserves the correlation structure of both data and input, while eliminating the relationship between the two signals (Goldfine et al., 2013), and the resulting threshold corresponds to an effective *p* ~ 0.01.

### Temporal mixture fit

The temporal mixture model illustrated in Figure 5 was as follows. The LFP RF *g*^*e*^_τ, *r*, θ, *o*_ measured on electrode *e* was assumed to be a noisy version of the underlying RF *f*^*e*^_τ, *r*, θ, *o*_. The underlying RF was given by a mixture of a shared component *p*_*r*, θ, *o*_ and a component specific to the electrode *q*^*e*^_*r*, θ, *o*_:

(5) $$f_{\tau ,\;r,\;\theta ,\;o}^{e}=a_{\tau }^{e}\;p_{r,\;\theta ,\;o}+b_{\tau }^{e}q_{r,\;\theta ,\;o}^{e}$$

The shared component was unconstrained while the specific component was a Gaussian in space modulated by orientation:

(6) $$q_{r,\;\theta ,\;o}^{e}=v_{o}^{e}G_{r}^{e}(r_{0},\;\sigma_{r})G_{\theta }^{e}(\theta_{0},\;\sigma_{\theta })$$

The temporal mixture model was fit using an iterative least-squares algorithm to minimize the mismatch between *f*^*e*^_τ, *r*, θ, *o*_ and *g*^*e*^_τ, *r*, θ, *o*_, which was estimated by reverse correlation. The shared component was initialized to the mean of all RFs at *t* = 90 ms for array 1 and *t* = 200 ms for array 2. *a*^*e*^_τ_ was then set by least-squares on the assumption that *b*^*e*^_τ_ = 0. Then the following steps were alternatively repeated until convergence:

1. The residual *g*^*e*^_τ, *r*, θ, *o*_ − *a*^*e*^_τ_*p*_*r*, θ, *o*_ was reshaped into a matrix with {*e*, τ} in one dimension and {*r*,θ,*o*} along the second dimension. The first singular values of this matrix were used to determine *b*^*e*^_τ_ and *q*^*e*^_*r*, θ, *o*_. *q*^*e*^_*r*, θ, *o*_ was then fit to Equation (6).
2. The first singular values of *g*^*e*^_τ, *r*, θ, *o*_ − *b*^*e*^_τ_*q*^*e*^_*r*, θ, *o*_ were used to determine *a*^*e*^_τ_ and *p*_*r*, θ, *o*_.

Once the temporal mixture model was fit, we extracted *f*^*e*^_τ, *r*, θ, *o*_ for each electrode and time lag and fit the reconstructed RF as an orientation filter multiplied by a Gaussian spatial envelope. The parameters determined through this process are plotted in Figures 6 and 8.

### Estimation of the integration radius of the LFP

We applied the method of Xing et al. (2009) to estimate the integration radius of the LFP on the cortical surface σ_cLFP_:

(7) $$\sigma_{c\text{LFP}}={\left[m^{2}(\sigma_{v\text{LFP}}^{2}-\sigma_{v\text{MUA}}^{2})+\sigma_{c\text{MUA}}^{2}\right]}^{2}$$

σ_vLFP_ and σ_vMUA_ correspond to the size of LFP and MUA RFs in visual coordinates, respectively, and σ_cMUA_ is the integration radius of the MUA in cortical coordinates. We used σ_cMUA_ = 100 μm (Xing et al., 2009) and estimated (σ^2^_vLFP_ − σ^2^_vMUA_) by taking the 20% trimmed mean of this quantity for electrodes where we could measure both LFP and MUA receptive fields.

The cortical magnification factor *m*, measured in millimeters per unit of visual space, captures the change in visual coordinates that corresponds to a unit change in position on the cortical surface. Thus, the local cortical magnification factor corresponds to the inverse of the magnitude of the gradient of the visual quantity measured (log eccentricity or polar angle in our case).

Unfortunately, estimating the magnitude of the gradient of the retinotopies of the MUAs directly is infeasible due to missing measurements. The measured retinotopies were too irregular to be fit reliably with a simple surface such as a plane. Instead, we obtained a smoothed estimate of log eccentricity and polar angle using Gaussian Process Regression (Rasmussen and Williams, 2006). We used the parameters suggested by the GPML for Matlab toolbox manual (Rasmussen and Williams, 2006; Gaussian likelihood, isometric squared exponential covariance, linear + constant mean function, marginal likelihood optimization for hyperparameters, exact inference).

We then computed the magnitude of the gradients of the smoothed surfaces, took their average across the surfaces, and inverted them to obtain the cortical magnification factor for log eccentricity and polar angle for each array.

### Orientation and temporal selectivity

We evaluated the orientation selectivity of the retinotopic component of the LFP and the MUA by computing the circular variance of the orientation selectivity coefficients [*v*_*o*_ in Equations (4) and (6)] as follows (Ringach et al., 2002):

(8) $$CV=1-\frac{\left|\sum_{\theta }v_{\theta }\exp(2i\theta )\right|}{\sum_{\theta }\left|v_{\theta }\right|}$$

For the non-retinotopic component of the LFP, the same formula was used, with *v*_*o*_ being estimated from the first singular vector of the SVD of the spatial-orientation filter.

We used the time mixture parameters *b*_τ_ (Equation 5) as an estimate of the temporal selectivity of the retinotopic component of the LFP (Figures 11E,F). For the MUA, we opted to take the first singular vector of (spatial-orientation)-temporal filters estimated by reverse correlation as an estimate of the temporal filters. These are more directly comparable to *b*_τ_ than *u*_τ_ in Equation (4), since *u*_τ_ corrects for the slight auto-correlation in the stimulus while *b*_τ_, being ultimately based on a reverse correlation estimate, does not.

### Conflict of interest statement

The authors declare that the research was conducted in the absence of any commercial or financial relationships that could be construed as a potential conflict of interest.

## References

[B1] Ahrens M. B. Paninski L. Sahani M. (2008). Inferring input nonlinearities in neural encoding models. Network 19, 35–67 10.1080/09548980701813936 18300178

[B2] Anastassiou C. A. Perin R. Markram H. Koch C. (2011). Ephaptic coupling of cortical neurons. Nat. Neurosci. 14, 217–223 10.1038/nn.2727 21240273

[B3] Andretic R. Van Swinderen B. Greenspan R. J. (2005). Dopaminergic modulation of arousal in Drosophila. Curr. Biol. 15, 1165–1175 10.1016/j.cub.2005.05.025 16005288

[B4] Atallah B. V. Scanziani M. (2009). Instantaneous modulation of gamma oscillation frequency by balancing excitation with inhibition. Neuron 62, 566–577 10.1016/j.neuron.2009.04.027 19477157 PMC2702525

[B5] Averbeck B. B. Latham P. E. Pouget A. (2006). Neural correlations, population coding and computation. Nat. Rev. Neurosci. 7, 358–366 10.1038/nrn1888 16760916

[B6] Bauer R. Brosch M. Eckhorn R. (1995). Different rules of spatial summation from beyond the receptive field for spike rates and oscillation amplitudes in cat visual cortex. Brain Res. 669, 291–297 10.1016/0006-8993(94)01273-K 7712185

[B7] Bedard C. Kröger H. Destexhe A. (2006). Model of low-pass filtering of local field potentials in brain tissue. Phys. Rev. E 73:051911 10.1103/PhysRevE.73.051911 16802971

[B8] Belitski A. Gretton A. Magri C. Murayama Y. Montemurro M. A. Logothetis N. K. (2008). Low-frequency local field potentials and spikes in primary visual cortex convey independent visual information. J. Neurosci. 28, 5696–5709 10.1523/JNEUROSCI.0009-08.2008 18509031 PMC6670798

[B9] Berens P. Keliris G. A. Ecker A. S. Logothetis N. K. Tolias A. S. (2008). Feature selectivity of the gamma-band of the local field potential in primate primary visual cortex. Front. Neurosci. 2, 199–207 10.3389/neuro.01.037.2008 19225593 PMC2622750

[B10] Blasdel G. G. Fitzpatrick D. (1984). Physiological organization of layer 4 in macaque striate cortex. J. Neurosci. 4, 880–895 6200586 10.1523/JNEUROSCI.04-03-00880.1984 PMC6564839

[B11] Buzsáki G. Anastassiou C. A. Koch C. (2012). The origin of extracellular fields and currents—EEG, ECoG, LFP and spikes. Nat. Rev. Neurosci. 13, 407–420 10.1038/nrn3241 22595786 PMC4907333

[B12] Carandini M. Ferster D. (2000). Membrane potential and firing rate in cat primary visual cortex. J. Neurosci. 20, 470–484 10627623 10.1523/JNEUROSCI.20-01-00470.2000 PMC6774139

[B13] Castro J. B. Kandler K. (2010). Changing tune in auditory cortex. Nat. Neurosci. 13, 271 10.1038/nn0310-271 20177415 PMC2989898

[B14] De Boer E. Kuyper P. (1968). Triggered correlation. IEEE Trans. Biomed. Eng. 15, 169–179 5667803 10.1109/tbme.1968.4502561

[B15] Eggermont J. J. Munguia R. Pienkowski M. Shaw G. (2011). Comparison of LFP-based and spike-based spectro-temporal receptive fields and cross-correlation in cat primary auditory cortex. PLoS ONE 6:e20046 10.1371/journal.pone.0020046 21625385 PMC3100317

[B16] Freeman J. Brouwer G. J. Heeger D. J. Merriam E. P. (2011). Orientation decoding depends on maps, not columns. J. Neurosci. 31, 4792–4804 10.1523/JNEUROSCI.5160-10.2011 21451017 PMC3086281

[B17] Freeman W. (2007). Hilbert transform for brain waves. Scholarpedia 2, 1338

[B18] Fries P. Reynolds J. H. Rorie A. E. Desimone R. (2001). Modulation of oscillatory neuronal synchronization by selective visual attention. Science 291, 1560–1563 10.1126/science.291.5508.1560 11222864

[B19] Fries P. Womelsdorf T. Oostenveld R. Desimone R. (2008). The effects of visual stimulation and selective visual attention on rhythmic neuronal synchronization in macaque area V4. J. Neurosci. 28, 4823–4835 10.1523/JNEUROSCI.4499-07.2008 18448659 PMC3844818

[B20] Ghose G. M. Ts'O D. Y. (1997). Form processing modules in primate area V4. J. Neurophysiol. 77, 2191–2196 9114265 10.1152/jn.1997.77.4.2191

[B21] Gieselmann M. A. Thiele A. (2008). Comparison of spatial integration and surround suppression characteristics in spiking activity and the local field potential in macaque V1. Eur. J. Neurosci. 28, 447–459 10.1111/j.1460-9568.2008.06358.x 18702717

[B22] Goldfine A. M. Bardin J. C. Noirhomme Q. Fins J. J. Schiff N. D. Victor J. D. (2013). Reanalysis of “Bedside detection of awareness in the vegetative state: a cohort study”. Lancet 381, 289–291 10.1016/S0140-6736(13)60125-7 23351802 PMC3641526

[B23] Gray C. M. Singer W. (1989). Stimulus-specific neuronal oscillations in orientation columns of cat visual cortex. Proc. Natl. Acad. Sci. U.S.A. 86, 1698–1702 2922407 10.1073/pnas.86.5.1698 PMC286768

[B24] Gregoriou G. G. Gotts S. J. Zhou H. Desimone R. (2009). High-frequency, long-range coupling between prefrontal and visual cortex during attention. Science 324, 1207–1210 10.1126/science.1171402 19478185 PMC2849291

[B25] Henrie J. A. Shapley R. (2005). LFP power spectra in V1 cortex: the graded effect of stimulus contrast. J. Neurophysiol. 94, 479–490 10.1152/jn.00919.2004 15703230

[B26] Hodgkin A. L. Huxley A. F. (1952). A quantitative description of membrane current and its application to conduction and excitation in nerve. J. Physiol. 117, 500 12991237 10.1113/jphysiol.1952.sp004764 PMC1392413

[B27] Hubel D. H. Wiesel T. N. (1977). Ferrier lecture: functional architecture of macaque monkey visual cortex. Proc. R. Soc. Lond. Ser. B Biol. Sci. 198, 1–59 20635 10.1098/rspb.1977.0085

[B28] Hwang E. J. Andersen R. A. (2011). Effects of visual stimulation on LFPs, spikes, and LFP-spike relations in PRR. J. Neurophysiol. 105, 1850–1860 10.1152/jn.00802.2010 21307325 PMC3075288

[B29] Jia X. Smith M. A. Kohn A. (2011). Stimulus selectivity and spatial coherence of gamma components of the local field potential. J. Neurosci. 31, 9390–9403 10.1523/JNEUROSCI.0645-11.2011 21697389 PMC3133446

[B30] Kajikawa Y. Schroeder C. E. (2011). How local is the local field potential? Neuron 72, 847–858 10.1016/j.neuron.2011.09.029 22153379 PMC3240862

[B31] Katzner S. Nauhaus I. Benucci A. Bonin V. Ringach D. L. Carandini M. (2009). Local origin of field potentials in visual cortex. Neuron 61, 35–41 10.1016/j.neuron.2008.11.016 19146811 PMC2730490

[B32] Khawaja F. A. Tsui J. M. G. Pack C. C. (2009). Pattern motion selectivity of spiking outputs and local field potentials in macaque visual cortex. J. Neurosci. 29, 13702–13709 10.1523/JNEUROSCI.2844-09.2009 19864582 PMC6665006

[B33] Koch C. (1999). Biophysics of Computation: Information Processing in Single Neurons. New York, NY: Oxford University Press

[B34] Kreiman G. Hung C. P. Kraskov A. Quiroga R. Q. Poggio T. DiCarlo J. J. (2006). Object selectivity of local field potentials and spikes in the macaque inferior temporal cortex. Neuron 49, 433–445 10.1016/j.neuron.2005.12.019 16446146

[B35] Lashgari R. Li X. Chen Y. Kremkow J. Bereshpolova Y. Swadlow H. A. (2012). Response properties of local field potentials and neighboring single neurons in awake primary visual cortex. J. Neurosci. 32, 11396–11413 10.1523/JNEUROSCI.0429-12.2012 22895722 PMC3436073

[B36] Liebe S. Logothetis N. K. Rainer G. (2011). Dissociable effects of natural image structure and color on LFP and spiking activity in the lateral prefrontal cortex and extrastriate visual area V4. J. Neurosci. 31, 10215–10227 10.1523/JNEUROSCI.1791-10.2011 21752998 PMC6623071

[B37] Lindén H. Tetzlaff T. Potjans T. C. Pettersen K. H. Grün S. Diesmann M. (2011). Modeling the spatial reach of the LFP. Neuron 72, 859–872 10.1016/j.neuron.2011.11.006 22153380

[B38] Liu J. Newsome W. T. (2006). Local field potential in cortical area MT: stimulus tuning and behavioral correlations. J. Neurosci. 26, 7779–7790 10.1523/JNEUROSCI.5052-05.2006 16870724 PMC6674213

[B39] Marmarelis P. Z. Marmarelis V. Z. (1978). Analysis of Physiological Systems: The White-Noise Approach. New York, NY: Plenum Press

[B40] Milstein J. Mormann F. Fried I. Koch C. (2009). Neuronal shot noise and Brownian 1/f2 behavior in the local field potential. PLoS ONE 4:e4338 10.1371/journal.pone.0004338 19190760 PMC2629847

[B41] Motter B. C. (2009). Central V4 receptive fields are scaled by the V1 cortical magnification and correspond to a constant-sized sampling of the V1 surface. J. Neurosci. 29, 5749–5757 10.1523/JNEUROSCI.4496-08.2009 19420243 PMC2694050

[B42] Mukamel R. Gelbard H. Arieli A. Hasson U. Fried I. Malach R. (2005). Coupling between neuronal firing, field potentials, and FMRI in human auditory cortex. Science 309, 951–954 10.1126/science.1110913 16081741

[B43] Nelson M. J. Pouget P. Nilsen E. A. Patten C. D. Schall J. D. (2008). Review of signal distortion through metal microelectrode recording circuits and filters. J. Neurosci. Methods 169, 141–157 10.1016/j.jneumeth.2007.12.010 18242715 PMC2292115

[B44] Nir Y. Fisch L. Mukamel R. Gelbard-Sagiv H. Arieli A. Fried I. (2007). Coupling between neuronal firing rate, gamma LFP, and BOLD fMRI is related to interneuronal correlations. Curr. Biol. 17, 1275–1285 10.1016/j.cub.2007.06.066 17686438

[B45] Ohki K. Chung S. Ch'ng Y. H. Kara P. Reid R. C. (2005). Functional imaging with cellular resolution reveals precise micro-architecture in visual cortex. Nature 433, 597–603 10.1038/nature03274 15660108

[B46] Ohki K. Chung S. Kara P. Hübener M. Bonhoeffer T. Reid R. C. (2006). Highly ordered arrangement of single neurons in orientation pinwheels. Nature 442, 925–928 10.1038/nature05019 16906137

[B47] Rasch M. J. Gretton A. Murayama Y. Maass W. Logothetis N. K. (2008). Inferring spike trains from local field potentials. J. Neurophysiol. 99, 1461–1476 10.1152/jn.00919.2007 18160425

[B48] Rasmussen C. E. Williams C. K. I. (2006). Gaussian Processes for Machine Learning. Cambridge, MA: MIT press

[B49] Ray S. Maunsell J. H. R. (2011). Different origins of gamma rhythm and high-gamma activity in macaque visual cortex. PLoS Biol. 9:e1000610 10.1371/journal.pbio.1000610 21532743 PMC3075230

[B50] Ringach D. L. Shapley R. M. Hawken M. J. (2002). Orientation selectivity in macaque V1: diversity and laminar dependence. J. Neurosci. 22, 5639–5651 12097515 10.1523/JNEUROSCI.22-13-05639.2002 PMC6758222

[B51] Supèr H. Roelfsema P. R. (2005). Chronic multiunit recordings in behaving animals: advantages and limitations, in Progress in Brain Research, ed van Pelt M. K. J. (Amsterdam: Elsevier), 263–282 10.1016/S0079-6123(04)47020-4 15581712

[B52] Tsui J. M. G. Pack C. C. (2011). Contrast sensitivity of MT receptive field centers and surrounds. J. Neurophysiol. 106, 1888–1900 10.1152/jn.00165.2011 21753021

[B53] Van Swinderen B. Nitz D. A. Greenspan R. J. (2004). Uncoupling of brain activity from movement defines arousal states in Drosophila. Curr. Biol. 14, 81–87 10.1016/j.cub.2003.12.057 14738728

[B54] Wood S. (2006). Generalized Additive Models: An Introduction with R. University of Bath, England: Chapman and Hall Available online at: http://www.crcpress.com/product/isbn/9781584884743 [Accessed February 7, 2013].

[B55] Xing D. Yeh C.-I. Shapley R. M. (2009). Spatial spread of the local field potential and its laminar variation in visual cortex. J. Neurosci. 29, 11540–11549 10.1523/JNEUROSCI.2573-09.2009 19759301 PMC2910581

[B56] Zanos T. P. Mineault P. J. Monteon J. A. Pack C. C. (2011a). Functional connectivity during surround suppression in macaque area V4. Conf. Proc. IEEE Eng. Med. Biol. Soc. 2011, 3342–3345 10.1109/IEMBS.2011.6090906 22255055

[B57] Zanos T. P. Mineault P. J. Pack C. C. (2011b). Removal of spurious correlations between spikes and local field potentials. J. Neurophysiol. 105, 474–486 10.1152/jn.00642.2010 21068271

